# NVP-BEZ235 Inhibits Renal Cell Carcinoma by Targeting TAK1 and PI3K/Akt/mTOR Pathways

**DOI:** 10.3389/fphar.2021.781623

**Published:** 2022-01-10

**Authors:** Bihui Li, Xing Zhang, Qianyao Ren, Li Gao, Jing Tian

**Affiliations:** ^1^ Guangxi Health Commission Key Laboratory of Tumor Immunology and Receptor-Targeted Drug Basic Research, Guilin Medical University, Guilin, China; ^2^ Department of Oncology, The Second Affiliated Hospital of Guilin Medical University, Guilin, China; ^3^ Department of Urinary Surgery, The First Affiliated Hospital of Guilin Medical University, Guilin, China

**Keywords:** renal cancer, NVP-BEZ235, PI3K, mTOR, TAK1

## Abstract

In spite of the promising *in vitro* and preclinical results, dual PI3K/Akt/mTOR inhibitor NVP-BEZ235, and ATP-competitive mTOR inhibitor PP242 both failed to confirm their inhibitory efficacy against renal cell carcinoma (RCC) in clinical settings. Therefore, a better understanding of the molecular mechanism is essential so as to provide possibilities for their use in combination with other agents. In present study, RCC cell lines (UMRC6, 786-0 and UOK121) were treated with NVP-BEZ235, PP242 or Rapamycin, an mTOR complex 1 (mTORC1)-specific inhibitor. They all suppressed cell proliferation and invasion, induced apoptosis and cell cycle arrest, and the effects were in the order of NVP-BEZ235 > PP242 > Rapamycin. Accordingly, the marked and sustained decrease in speckle-type POZ protein (SPOP) expression and phosphorylation of Akt and mTOR kinases was observed in RCC cells treated with NVP-BEZ235 and PP242, whereas only potent inhibition of mTOR activity was induced in Rapamycin-treated cells. In considering the overactivation of c-Jun and IκB-α in human renal tumor tissue, we next investigated the role of JNK and IKK pathways in the response of RCC cells to these compounds. First of all, transforming growth factor β activated kinase 1 (TAK1)-dependent activation of JNK/ (activator protein-1) AP-1 axis in RCC cells was proved by the repression of AP-1 activity with TAK1 or JNK inhibitor. Second, the profound inhibition of TAK1/JNK/AP-1 pathway was demonstrated in RCC cells treated with NVP-BEZ235 or PP242 but not Rapamycin, which is manifested as a reduction in activity of TAK1, c-Jun and AP-1. Meanwhile, subsequent to TAK1 inactivation, the activation of IκB-α was also reduced by NVP-BEZ235 and PP242. Likewise, *in vivo*, treatment with NVP-BEZ235 and PP242 suppressed the growth of xenografts generated from 786-0 and A498 cells, along with decreased expression of phospho-TAK1, phospho-c-Jun, and phospho-IκB-α. In contrast, Rapamycin elicited no significant inhibitory effects on tumor growth and phosphorylation of TAK1, c-Jun and IκB-α. We conclude that besides PI3K/Akt/mTOR signaling, NVP-BEZ235, and PP242 simultaneously target TAK1-dependent pathways in RCC cells. Notably, these effects were more marked in the presence of NVP-BEZ235 than PP242, indicating the potential application of NVP-BEZ235 in combination therapy for RCC.

## Introduction

Renal cell carcinomas (RCC) is the most frequent type of cancer originating from the kidney parenchyma (Inamura, 2017; Pietropaolo et al., 2019). Every year, approximately more than 200,000 individuals are newly diagnosed with RCC worldwide (Bray et al., 2018). Unfortunately, RCC is largely resistant to traditional chemotherapy, radiation, or hormonal therapy, leading to over 100,000 deaths per year (Linehan and Ricketts, 2017). Based on the growing understanding of the underlying molecular pathways in RCC, the drugs targeting the PI3K/Akt/mammalian target of rapamycin (mTOR) have been discovered and evaluated in clinical trials, but their clinical efficacy is limited by resistance, toxicity, and poor tolerability (Guo et al., 2015). To overcome these limitations, the combination with other therapeutic drugs has been attracting the attention of researchers. This requires further elucidation of the mechanism of these PI3K/AKT/mTOR inhibitors.

As is known, activated PI3K/Akt/mTOR signaling pathway is critical for many important cellular processes including proliferation, growth and survival (Chiarini et al., 2015; Kaur and Sharma, 2017). Rapamycin and its analogs are the first generation of mTOR inhibitors that selectively inhibit the activity of mTOR complex 1 (mTORC1), a multiprotein complex containing mTOR (Xu et al., 2016). Although Rapamycin raised the possibility of developing antitumor agent targeting PI3K/Akt/mTOR pathway, results have demonstrated that inhibiting mTORC1 induces feedback activation of the upstream PI3K/Akt pathway and thereby counteracts the anticancer efficacy (Osawa et al., 2019; Roskoski, 2019). Then new generations of agents targeting both mTORC1 and mTOR complex 2 (mTORC2) have been developed, for example PP242 and NVP-BEZ235. They both displayed anti-cancer activity against various types of cancer cells including RCC cells (Yang et al., 2013). What is more, besides inhibiting mTOR, NVP-BEZ235 could bind directly to the ATP-binding domain of PI3K and block PI3K-dependent Akt activation (Alqurashi et al., 2018). It may be hypothesized that dual PI3K/mTOR inhibitor NVP-BEZ235 is a more potent antitumor agent against RCC than the mTOR kinase inhibitor PP242 and selective mTORC1 inhibitor Rapamycin. Nevertheless, to our knowledge, there has been no study comparing the anticancer effects of NVP-BEZ235, PP242, and Rapamycin, not to mention deep investigation of the exact mechanism behind their difference.

Notably, emerging evidence proved that PI3K/Akt/mTOR pathway correlates with a new form of nonapoptotic cell death, ferroptosis, that is distinct from known forms of cell death such as apoptosis, necroptosis, and necrosis (Yi et al., 2020). Ferroptosis is caused by an imbalance in redox homeostasis due to glutathione (GSH) depletion or inactivation of glutathione peroxidase 4 (GPX4) (Hirschhorn and Stockwell, 2019). The dysregulation of ferroptosis has been linked to various cancers (Mou et al., 2019). It was found that inhibition of mTORC1 could trigger the degradation of GPX4 protein, and finally promote ferroptosis in cancer cells (Lei et al., 2021). Accordingly, mTORC1 is also considered as a key ferroptosis modulator. Consequently, the specific inhibition of mTORC1 *via* Rapamycin was demonstrated to induce ferroptotic cancer cell death and inhibit tumorigenesis. Hence, exploring the relationship between mTOR inhibitors and ferroptosis may offer a new perspective for the mechanisms of mTOR inhibitors in RCC.

In this study, as expected, it was demonstrated that NVP-BEZ235 inhibits RCC cells growth *in vitro* and *in vivo*, better than PP242 and Rapamycin. In addition to PI3K/Akt/mTOR signaling pathway, NVP-BEZ235, and PP242 also suppressed the activation of TGF-β-associated kinase 1 (TAK1) and its downstream effectors including c-Jun and IκB-α. Moreover, the inhibition was more remarkable in NVP-BEZ235-treated RCC cells. These results indicated that the dual suppression of PI3K/Akt/mTOR and TAK1 pathways by NVP-BEZ235 and PP242 may link to their greater anticancer activity, and NVP-BEZ235 may be an optimal candidate as mTOR inhibitor for combination therapy in RCC.

## Materials and Methods

### Cell Lines and Reagents

Three human renal cell carcinoma cell lines, UMRC6 (from B Zbar, National Cancer Institute, Bethesda, MD, United States), 786-0 (from W Kaelin, Dana Farber Cancer Institute, Boston, MA, United States), and UOK121 cells (from J. Gnarra, Louisiana State University, Baton Rouge, LA, United States) have been described previously (An et al., 2013). HEK-293 cells were purchased from Shanghai cell bank of the Chinese Academy of Sciences (Shanghai, China). Cells were maintained in Dulbecco’s modified Eagle’s medium (DMEM; Invitrogen, Carlsbad, CA, United States) supplemented with 10% FBS and 1% penicillin-streptomycin at 37°C in a humidified atmosphere of 5% CO_2_. NVP-BEZ235 (Novartis Pharmaceuticals, Basel, Switzerland), PP242 (Sigma-Aldrich, St Louis, MO, United States), and Rapamycin (Santa Cruz Biotechnology, Santa Cruz, CA, United States) were solubilized in DMSO (*in vitro* assays) or a solution of 2% DMSO, 30% PEG 300, 5% Tween 80, and ddH2O (*in vivo* assays). Antibodies against Akt, phospho-Akt, TAK1, phospho-TAK1, IκB-α, phospho-IκB-α, c-Jun, and phospho-c-Jun were all purchased from Cell Signaling Technology (Beverly, MA, United States). Antibodies against phospho-mTOR, speckle-type POZ protein (SPOP), and β-actin were from Santa Cruz Biotechnology.

### Clinical Materials

The two RCC tissue microarrays were purchased from Fanpu Biotech, Inc. (Guilin, Guangxi, China). One chip consisted of 87 patients with RCC (65 cases of clear cell RCC and 22 cases of papillary RCC) and samples of normal tissues from 8 patients. The other one included 60 patients with RCC (47 cases of clear cell RCC and 13 cases of papillary RCC) and samples of normal tissues from 5 patients. The chips were heated at 60°C to melt the paraffin, washed in xylene, and hydrated through graded ethanols (100–70%). For antigen retrieval, the chips were heated in Tris-EDTA for 20 min, and then blocked with 10% goat serum. Phospho-c-Jun and phospho-IκB-α staining was performed by incubation with rabbit anti-phospho-c-Jun antibody (1:100) and rabbit anti-phospho-IκB-α antibody (1:250) at 4°C overnight, followed by incubation of secondary antibody for 1 h at room temperature. Finally, the chips were stained with DAB chromogen solution and counterstained with hematoxylin.

### Cell Viability Assays

The three RCC cell lines and HEK-293 cells were seeded in 96-well plates at a density of 1.5 × 10^3^ or 3.0 × 10^3^ per well. Cells were then treated with increasing doses (10, 100, 1,000 nM) of NVP-BEZ235, PP242, and Rapamycin for 48 h, or the equivalent amounts of DMSO as control. MTT (Sigma-Aldrich) was added to each well (1 mg/ml final concentration) and incubated at 37°C for 4 h. Then the plates were incubated with 100 μL of 10% SDS/0.01 N HCl. After incubation overnight, absorbance at 490 nM for each well was determined using a microplate reader (Bio-Rad, Sunnyvale, CA, United States). For colony formation assay, 786-0 and UOK121 cells were seeded in six-well plates with 500 cells per well, and cultured with 200 nM NVP-BEZ235, Rapamycin and PP242, or equivalent amounts of DMSO. Colonies were counted after 10–14 days of incubation.

### Flow Cytometry Assay

Cell poptosis was analyzed using FITC Annexin V Apoptosis Detection Kit (BD Biosciences, San Jose, CA, United States). Briefly, after exposure to 100 nM of NVP-BEZ235, PP242, or Rapamycin for 72 h, cells were harvested, washed twice with cold PBS and resuspended in binding buffer at a density of 1 × 10^6^ cells/ml. About 1 × 10^5^ cells were stained with annexin V-FITC and propidium iodide (PI) for 15 min at room temperature in the dark and analyzed using a Beckman FC-500 flow cytometer (Beckman Coulter, CA, United States). For cell cycle assay, cells were respectively treated with different doses (100 and 500 nM) of NVP-BEZ235, PP242, and Rapamycin for 48 h, or equivalent amounts of DMSO. Then cells were fixed with 70% alcohol at 4°C overnight and stained with PI (5 ng/ml) for 20 min at room temperature. The cell distribution at various cell cycle phases were determined by flow cytometry.

### Transwell Assay

First, the chamber membranes were coated using Matrigel (50 µg/ml). 2 × 10^5^ cells were added in the upper chambers with medium containing 1% FBS. The lower chamber was filled with DMEM containing 10% FBS with or without 200 nM of NVP-BEZ235, PP242, Rapamycin. After incubating for 48 h, cells invaded to the lower chamber were stained with 0.5% crystal violet, and counted using a microscope at 200 × magnification.

### Western Blot Assay

For time-dependent studies, cells were treated with 200 nM of NVP-BEZ235, PP242 and Rapamycin, or the equivalent amounts of DMSO as control. At different time points post-treatment, cells were harvested and lysed by incubation in lysis solution containing protease inhibitors. Equal amounts of total protein (20 or 40 µg) were separated by sodium dodecyl sulfate-polyacrylamide gel electrophoresis (SDS-PAGE), and transferred to polyvinylidene difluoride (PVDF) membrane (Millipore, Billerica, MA, United States). Membranes were probed with following primary antibodies: phospho- and total Akt, phospho-mTOR, SPOP, phospho- and total TAK1, phospho- and total c-Jun, phospho- and total IκB-α, β-actin. Band intensities were determined using ImageJ software (version 1.48; National Institutes of Health, Bethesda, MD, United States). For dose-dependent studies, cells were treated with increasing doses (10, 100, 500, 1,000 nM) of NVP-BEZ235, PP242, Rapamycin for 48 h. Protein was prepared and measured as described above. Data were expressed as ratio of phosphorylated component/total protein, p-mTOR/β-actin, or SPOP/β-actin.

### Luciferase Reporter Assay

For luciferase assay, RCC cell lines were transfected with the AP-1 or IκB-α reporter vector and a Renilla luciferase plasmid (Promega, Madison, WI, United States) as a control for transfection efficiency. To study whether AP-1 activation is regulated by TAK1/JNK pathway in RCC cells, the transfected cells were pretreated with TAK1-inhibitor 5Z-7-oxozeaenol (0, 50, 100, 200 nM) (Tocris Bioscience, Ellisville, MO, United States) or JNK-inhibitor II SP600125 (0, 2.5, 5, 10 µM) (Calbiochem, San Diego, CA, United States) for 1 h. Furthermore, to study the possible role of AP-1 and IκB-α in anticancer activities of NVP-BEZ235, PP242, and Rapamycin, the transfected cells were treated with these compounds (10, 100, 1,000 nM) for 24 h, or the equivalent amounts of DMSO as control. Luciferase activity was measured using a Dual-Glo^®^ Luciferase Assay System (Promega) according to the manufacturer’s protocol.

### Xenograft Model and Immunohistochemical Staining

Four-week old female BALB/c nude mice were purchased from the Animal Experiment Center of Guilin Medical University (Guilin, Guangxi, China). 786-0 and A498 cells were chosen for *in vivo* study. A498 cells were purchased from Institute of Biochemistry and Cell Biology of the Chinese Academy of Sciences (Shanghai, China). 1 × 10^7^ 786-0 or A498 cells were injected subcutaneously into nude mice. When the tumor xenografts reached around 300 mm^3^, mice were randomized into different groups (*n* = 6/group), and given NVP-BEZ235, PP242 or Rapamycin (15 mg/kg) every 2 days *via* oral gavage. Tumor size and mice weight were measured every other day. Mice were sacrificed after 28 days of treatment, and then the tumors were excised for histological examination. Tumor samples were fixed and embedded in paraffin. Then 5 μm tissues sections were deparaffinized and immunolabeled with antibodies against phospho-TAK1, phospho-IκB-α, and phospho-c-Jun. The intensity of staining versus background staining was visually determined under a light microscope. For the animal experiments, all procedures were approved by the Animal Research Ethics Committee of Guilin Medical University.

### Statistical Analysis

All data were expressed as mean ± standard deviation (SD). Statistical significance was determined using a one-way ANOVA followed by Tukey’s post hoc test using the SPSS statistical program (version 12.0; SPSS, Chicago, IL). A value of *p* < 0.05 was considered significant.

## Results

### Comparison of the Inhibitory Effects of NVP-BEZ235, PP242, and Rapamycin in RCC Cell Lines

**FIGURE 1:**
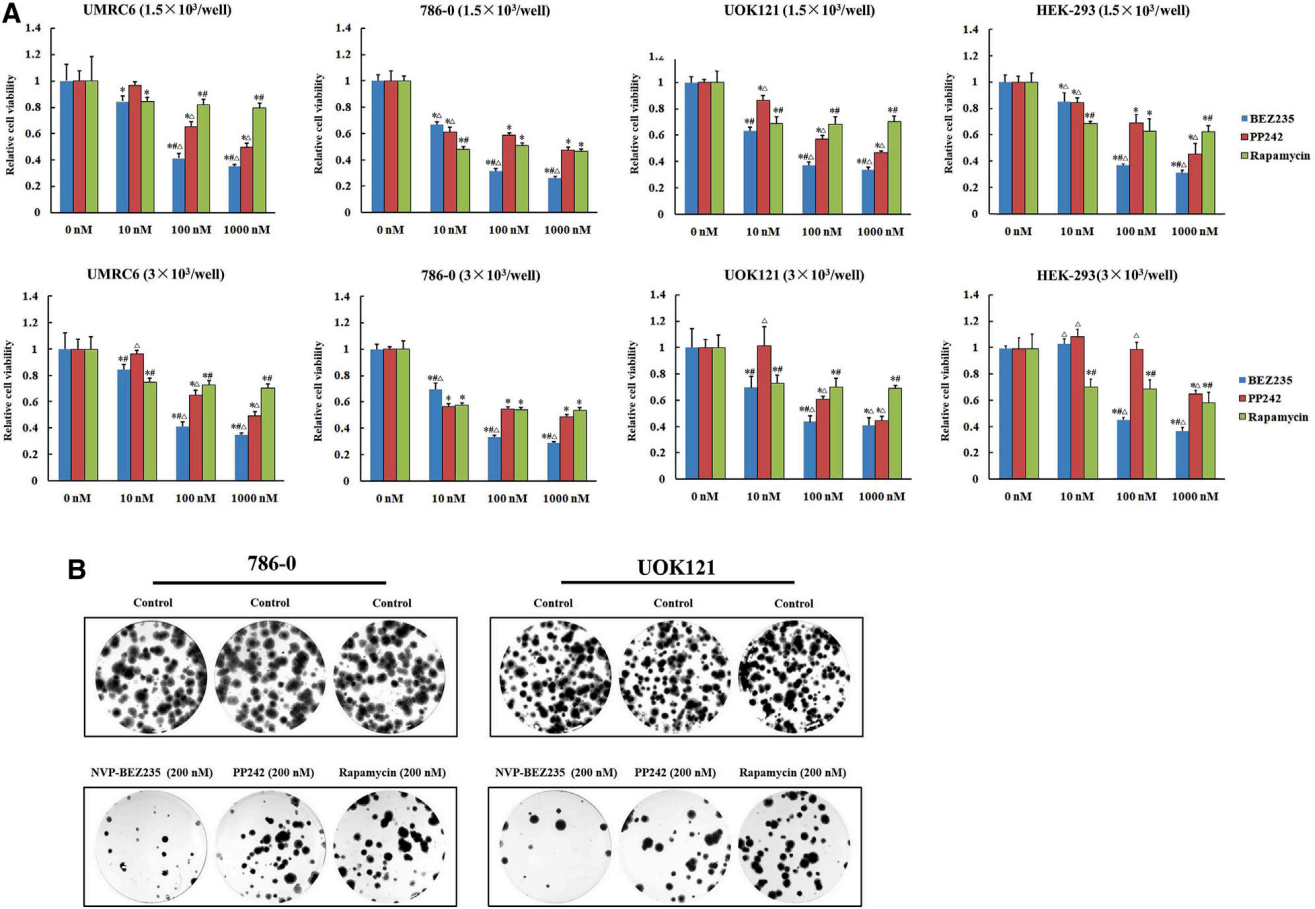
Suppression of RCC cells proliferation by NVP-BEZ235, PP242, and Rapamycin *in vitro*. **(A)** 1.5 × 10^3^ or 3.0 × 10^3^ RCC cells (UMRC6, 786-0, and UOK121) and HEK-293 cells were treated with increasing concentrations of NVP-BEZ235, PP242, and Rapamycin for 48 h. Cell viability was measured with MTT. DMSO-treated cells served as control (0 nM). All value are means ± SD of three replicates. *p < 0.05, versus the corresponding control (0 nM) group; #*p* < 0.05, versus the corresponding PP242 group; ∆*p* < 0.05, versus the corresponding Rapamycin group. **(B)** For colony formation assay, 500 cells of 786-0 and UOK121 were cultured in six-well plate, and cultured with 200 nM of NVP-BEZ235, PP242, and Rapamycin. After 10-14 days of incubation, colonies were stained with Giemsa stain, and counted. Results are representative of three independent experiments.

We here used three human renal cancer cell lines, UMRC6, 786-0, and UOK121, to access and compare the anticancer activities of NVP-BEZ235, PP242, and Rapamycin. It was found that the proliferation of all cell lines was significantly reduced by each compound (Figure 1A). Moreover, treatment with NVP-BEZ235 produced the greatest reduction in cell viability, followed by PP242 and Rapamycin. As control, the normal human embryonic kidney HEK-293 cells were also treated with NVP-BEZ235, PP242, and Rapamycin. Similar proliferation inhibition was induced by treatment with high concentrations of the three compounds, but not by 10 nM NVP-BEZ235 and PP242. It is indicated that at appropriate concentrations, NVP-BEZ235 and PP242 could exert anti-proliferative effects on RCC cells with less profound effect on normal cells. Likewise, the most marked decrease in numbers of colonies was observed in 786-0 and UOK121 cells treated with NVP-BEZ235 (Figure 1B).

**FIGURE 2:**
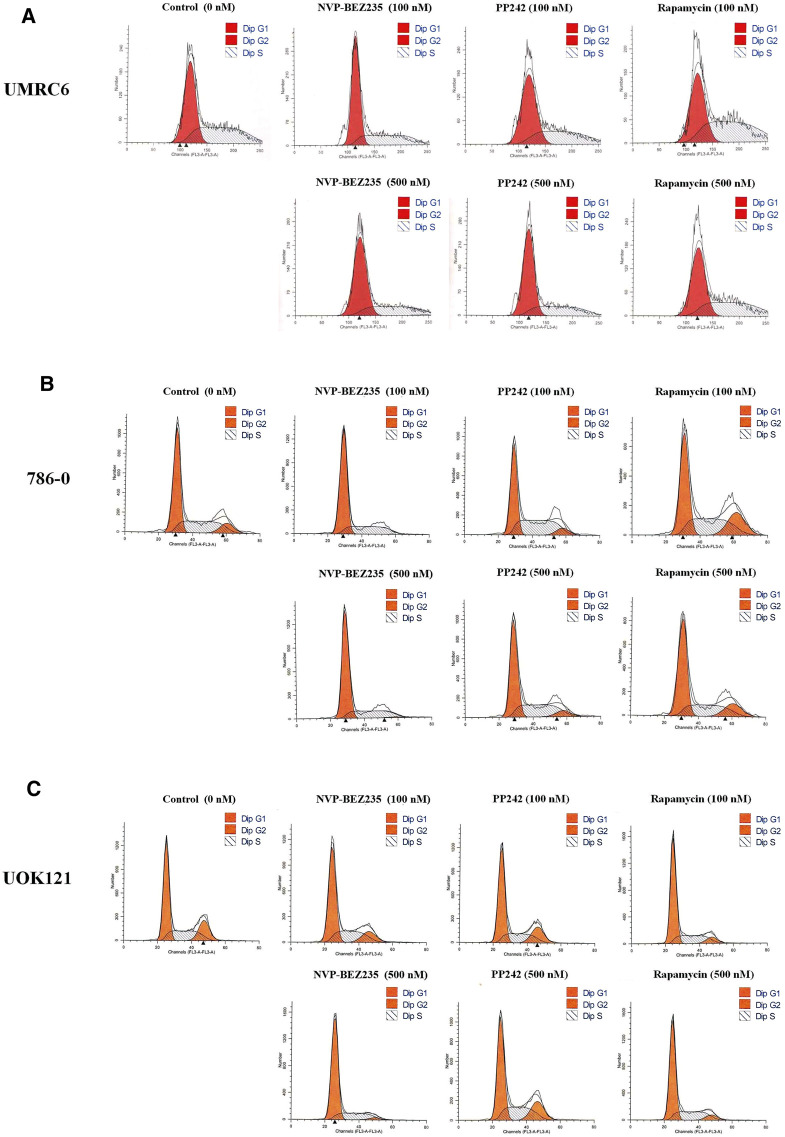
Treatment with NVP-BEZ235, PP242, and Rapamycin induced cell cycle arrest in RCC cells. **(A)** UMRC6, **(B)** 786-0, and **(C)** UOK121 cells were incubated with indicated concentrations of NVP-BEZ235, PP242, and Rapamycin for 72 h. DMSO-treated cells served as control (0 nM). The proportion of cells in different phases of the cell cycle was determined by flow cytometry.

**FIGURE 3:**
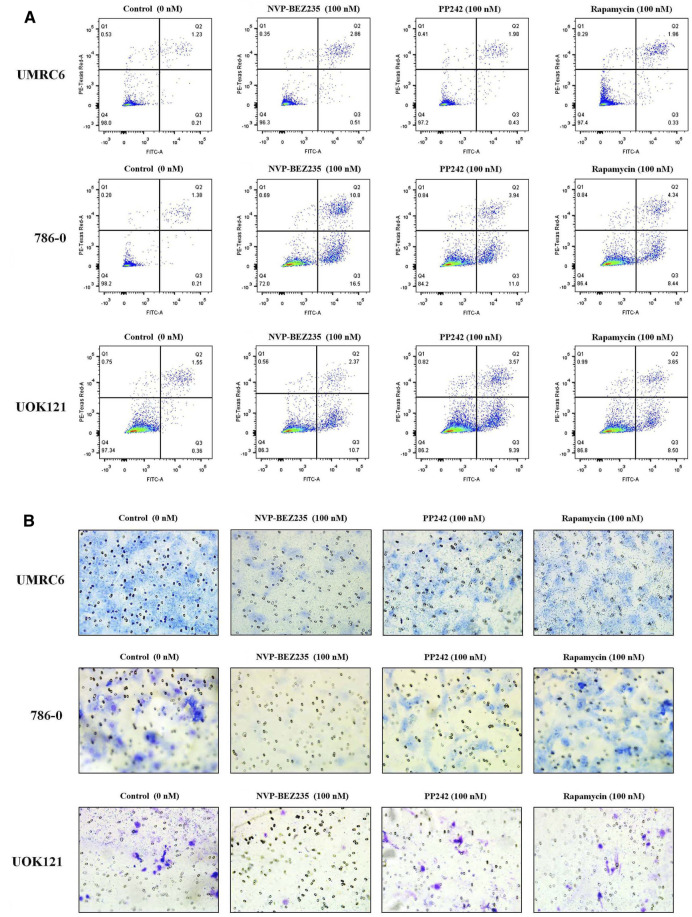
Treatment with NVP-BEZ235, PP242, and Rapamycin increased apoptosis and reduced tumor cell invasion in RCC cells. UMRC6, 786-0, and UOK121 cells were respectively incubated with 100 nM NVP-BEZ235, PP242, and Rapamycin for 48 h or 72 h. DMSO-treated cells served as control (0 nM). **(A)** The apoptotic cell death was quantified by flow cytometry with Annexin V-FITC and PI staining. **(B)** A transwell invasion assay was performed on RCC cells with NVP-BEZ235, PP242, or Rapamycin. After 48 h, the invasive cells were stained with crystal violet and counted (final magnification, 200×). Results are representative of three independent experiments.

Additional studies were done to address the antiproliferative mechanism of NVP-BEZ235, PP242, and Rapamycin. Flow cytometric analysis of the cell cycle distribution of RCC cells showed that the cells were partially blocked in the G1 phase in all cultures, especially in NVP-BEZ235-treated cell lines (Figure 2). Cells at the G1 phase increased from 54.54% in control group to 69.03% (500 nM NVP-BEZ235) in UMRC6 cells, from 54.76 to 71.79% in 786-0 cells, from 51.79 to 73.30% in UOK121 cells. Meanwhile, we observed that the percentage of apoptotic cells increased significantly in RCC cells when treated with the three compounds (Figure 3A). Also, NVP-BEZ235 possessed higher pro-apoptotic activity than PP242 and Rapamycin. These findings indicate that apoptosis induction and cell cycle arrest contribute to the growth inhibition of RCC cells by NVP-BEZ235, PP242, and Rapamycin. At the same time, these compounds significantly reduced cell migration in the order NVP-BEZ235 > PP242 > Rapamycin (Figure 3B).

### Inhibition of PI3K/Akt/mTOR Pathway Signaling in RCC Cell Lines by NVP-BEZ235, PP242, and Rapamycin

**FIGURE 4:**
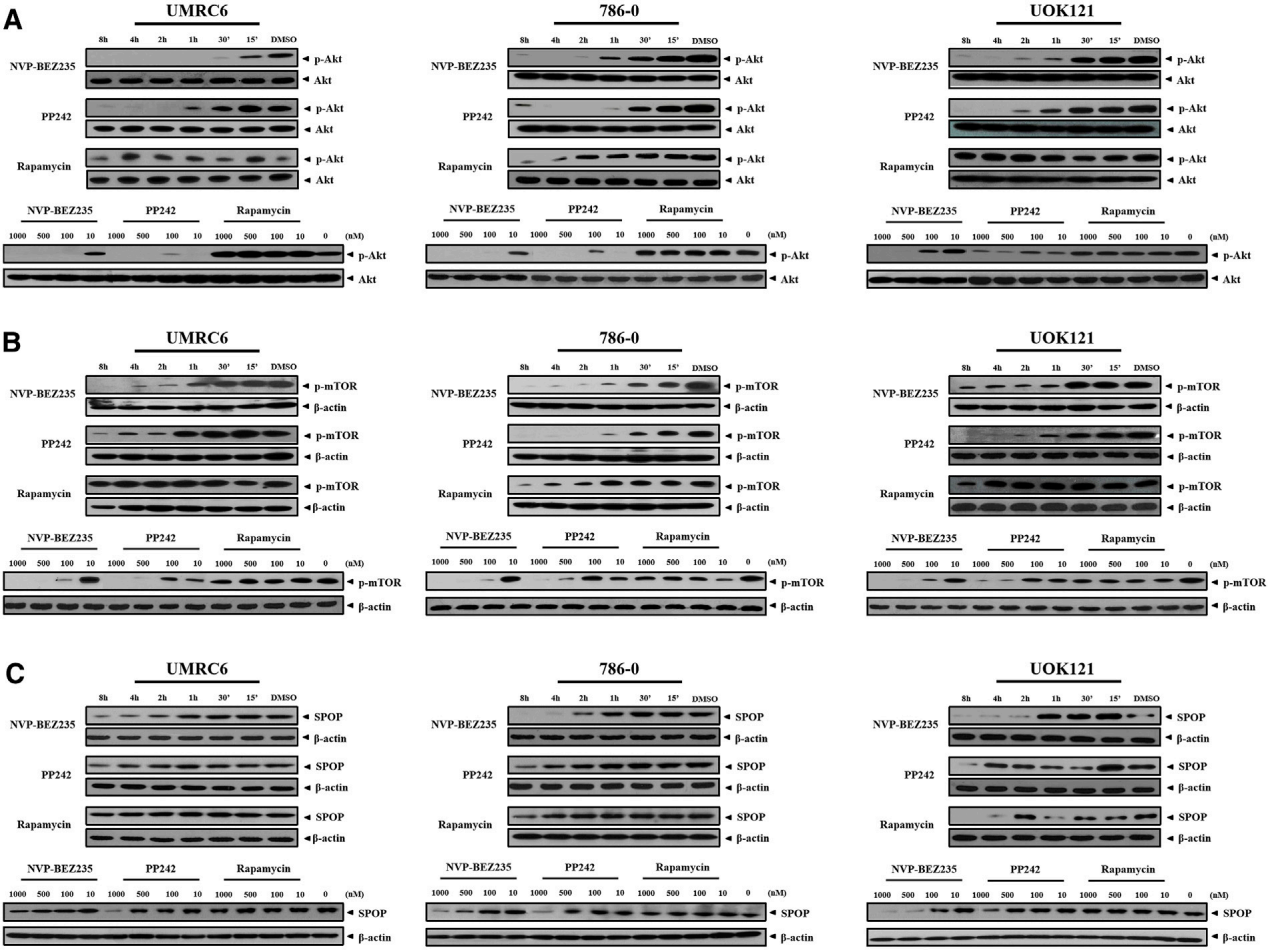
Dose- and time-dependent regulation of PI3K/Akt/mTOR pathway by NVP-BEZ235, PP242, and Rapamycin. RCC cells (UMRC6, 786-0, and UOK121) were exposed to increasing concentrations (10, 100, 500, and 1,000 nM) of NVP-BEZ235, PP242, and Rapamycin for 48 h, or 200 nM of these compounds for different time periods as indicated, after which the alteration of phosphorylation of **(A)** Akt, **(B)** mTOR, and **(C)** SPOP expression was examined by Western blot respectively. The relative amounts of p-Akt normalized to total Akt, p-mTOR, and SPOP normalized to β-actin, are shown. Cells harvested at 0 h or DMSO-treated cells (0 nM) served as control. Results are representative of three independent experiments.

NVP-BEZ235, PP242, and Rapamycin are inhibitors of PI3K/Akt/mTOR pathway. Consistent with previous studies, it was shown that NVP-BEZ235 and PP242 inhibited phosphorylation of Akt and mTOR in UMRC6, 786-0, and UOK121 cells, but Rapamycin only decreased p-mTOR expression in the three cell lines (Figures 4A,B; Supplementary Figure S1). On the contrary, Rapamycin induced feedback activation of Akt in UMRC6 cells, in coincidence with previous report (Osawa et al., 2019). Nevertheless, in UOK121 and 786-0 cells, Rapamycin produced no significant change in Akt phosphorylation, though there was an obvious decrease in p-Akt levels within the first 8 h after exposure in 786-0 cells. In view of the relationship between SPOP mutations and activation of PI3K pathway in cancers, we next detected the alteration of SPOP in RCC cells. NVP-BEZ235 and PP242 suppressed SPOP protein expression in RCC cell lines in a dose- and time-dependent manner, coincident with reduced Akt phosphorylation, whereas no obvious change was observed in Rapamycin-treated cells after 48 h of exposure (Figure 4C; Supplementary Figure S2A). Furthermore, in comparison with PP242, NVP-BEZ235 had a superior effect on reduction of SPOP expression and inactivation of PI3K/Akt/mTOR pathway, and this effect was more pronounced in 786-0 and UOK121 cells.

### Inhibition of TAK1/JNK/AP-1 and TAK1/IκB Kinase Pathways in RCC Cell Lines by NVP-BEZ235 and PP242 Rather Than Rapamycin

**FIGURE 5:**
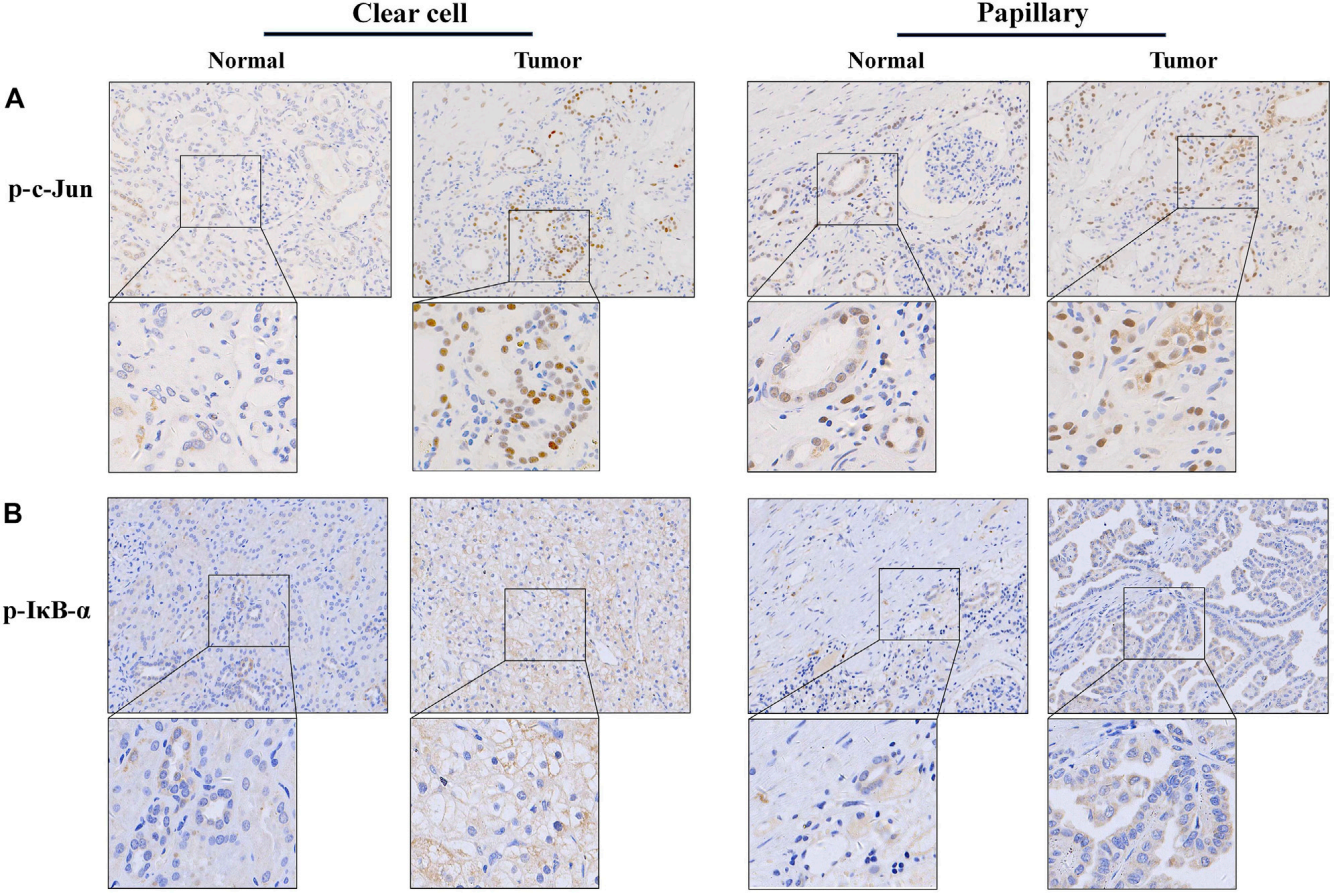
Immunohistochemistry analysis of phospho-c-Jun and phospho-IĸB-α expression in RCC tissue. A total of 147 RCC tissue samples were analyzed for stain localization and intensity. Immunohistochemistry was performed using polyclonal antibodies specific for p-c-Jun or p-IĸB-α. **(A)** phosphorylated c-Jun and **(B)** phosphorylated IĸB-α staining were mainly localized in the nucleus or cytoplasm, respectively. Their expression was both faint in the of normal renal tissue, and more conspicuous in the RCC tissue. (original magnification 200×, inset 400×).

**FIGURE 6:**
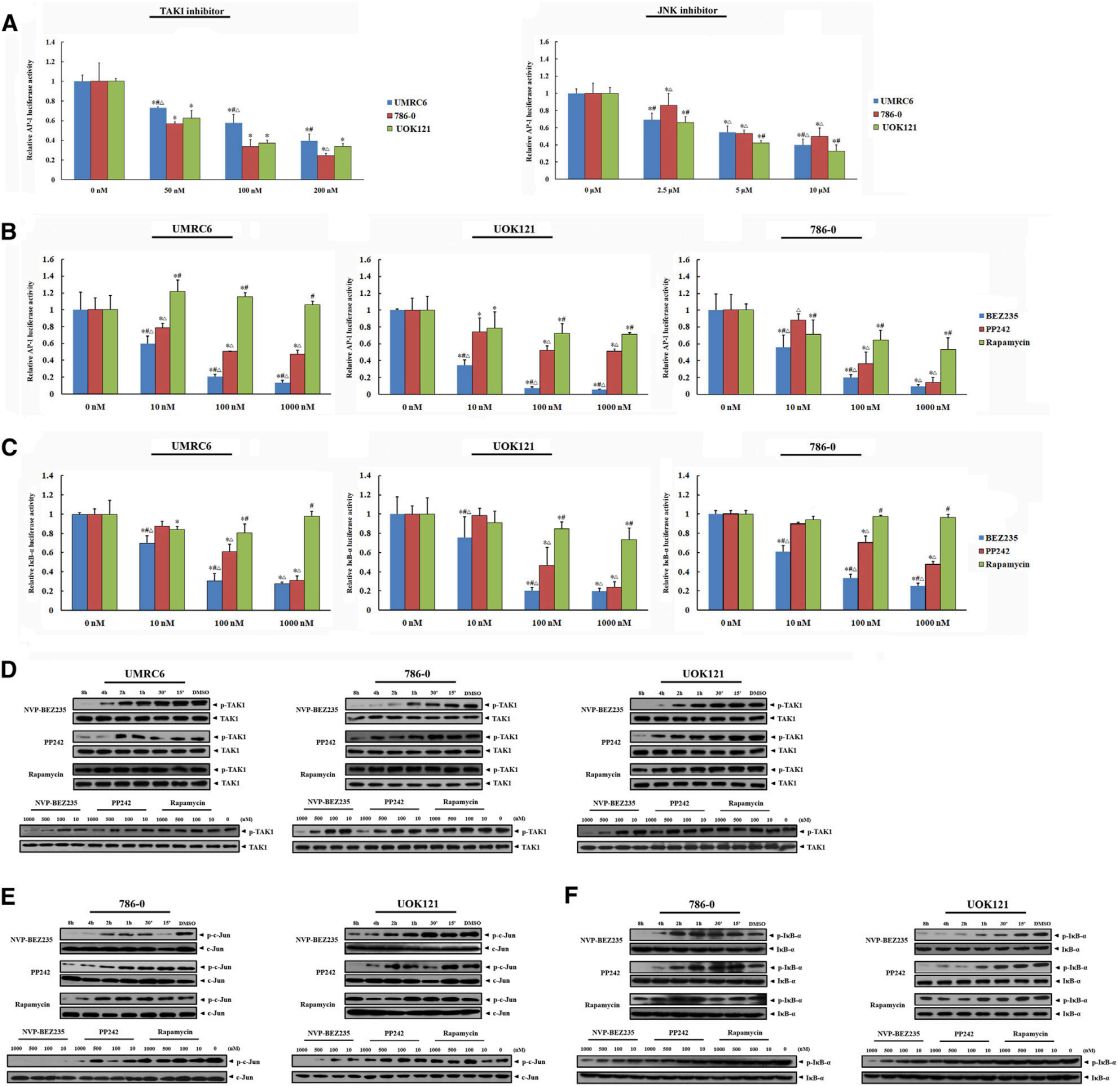
Inhibition of JNK/AP-1 and IKK pathways *via* TAK1 in RCC cell lines by NVP-BEZ235 and PP242. **(A)** Effect of JNK or TAK1 inhibition on AP-1 transcriptional activity in RCC cells assessed by luciferase reporter assay. UMRC6, 786-0, and UOK121 cells were transfected with AP-1 reporter plasmid and a Renilla-luciferase control plasmid, followed by incubation with TAK1-inhibitor or JNK-inhibitor for 24 h. Untreated cells served as control (0 μM). The AP-1-dependent. firefly luciferase activity was normalized to the Renilla luciferase activity as a transfection control. Next, the transfected RCC cells were incubated with various concentrations (10, 100, 1,000 nM) of NVP-BEZ235, PP242, or Rapamycin for 24 h. The **(B)** AP-1 activity and **(C)** IκB-α activity were measured by the ratio of firefly luciferase activity to Renilla luciferase activity. DMSO-treated cells (0 nM) served as control. All value are means ± SD of three replicates. *p < 0.05, versus the corresponding control (0 nM) group; #*p* < 0.05, versus the corresponding PP242 group; ∆*p* < 0.05, versus the corresponding Rapamycin group. Western blot assay further confirmed the inhibition of **(D)** TAK1, **(E)** JNK, and **(F)** IKK activation. RCC cells were incubated with 10, 100, 500, 1,000 nM of NVP-BEZ235, PP242 and Rapamycin for 48h, or 200 nm of these compounds for the time courses as indicated. The relative phosphorylation levels of TAK1, c-Jun and IκB-α were calculated and normalized to total protein. Cells harvested at 0 h or DMSO-treated cells (0 nM) served as control. Results are representative of three independent experiments.

In present study, the elevated phosphorylation levels of c-Jun and IκB-α were found in RCC tissue samples, including both clear cell and papillary renal cell carcinomas, confirming the participation of JNK and IKK activation in the development and progression of renal carcinoma (Figure 5). Thus, we here focused on TAK1, a member of the mitogen-activated protein kinase kinase kinase (MAPKKK) family that can function in the JNK and IKK pathways (An et al., 2013). Exposure of RCC cell lines to NVP-BEZ235 and PP242 reduced phosphorylation of TAK1 in a time- and dose-dependent manner (Figure 6D). Additionally, the specific inhibitors of TAK1 and JNK both dose-dependently provoked a reduction of AP-1 activity, confirming that AP-1 acts as downstream of TAK1, and JNK in RCC cells (Figure 6A). Correspondingly, the inhibition on TAK1 and JNK by NVP-BEZ235 and PP242 was accompanied with reduced AP-1 activity and inhibition of c-Jun phosphorylation (Figures 6B,E; Supplementary Figures S2B, S3A). On the other hand, although Rapamycin suppressed AP-1 activation in 786-0 and UOK121 cells, it failed to significantly inhibit phosphorylation of TAK1 and c-Jun in RCC cell lines, except for a temporary down-regulation of p-TAK1 and p-c-Jun expression. IκB-α is another downstream molecule of TAK1, and its phosphorylation is mainly mediated by IKK. Subsequent to decreased TAK1 phosphorylation, the activation of IκB-α was steadily and significantly inhibited by NVP-BEZ235 and PP242 rather than Rapamycin in RCC cells (Figures 6C,F; Supplementary Figure S3B). The decline trend of c-Jun/AP-1 activity, Akt, and IκB-α phosphorylation was much more marked in RCC cells treated with NVP-BEZ235. These results demonstrated that TAK1-dependent JNK and IKK signaling is involved in anti-cancer mechanism of NVP-BEZ235 and PP242 in renal cell carcinoma, which may be a new possible explanation to the different response of RCC cells to these compounds.

### Comparison of the Antitumor Activity of NVP-BEZ235, PP242, and Rapamycin in RCC Xenograft Models

**FIGURE 7:**
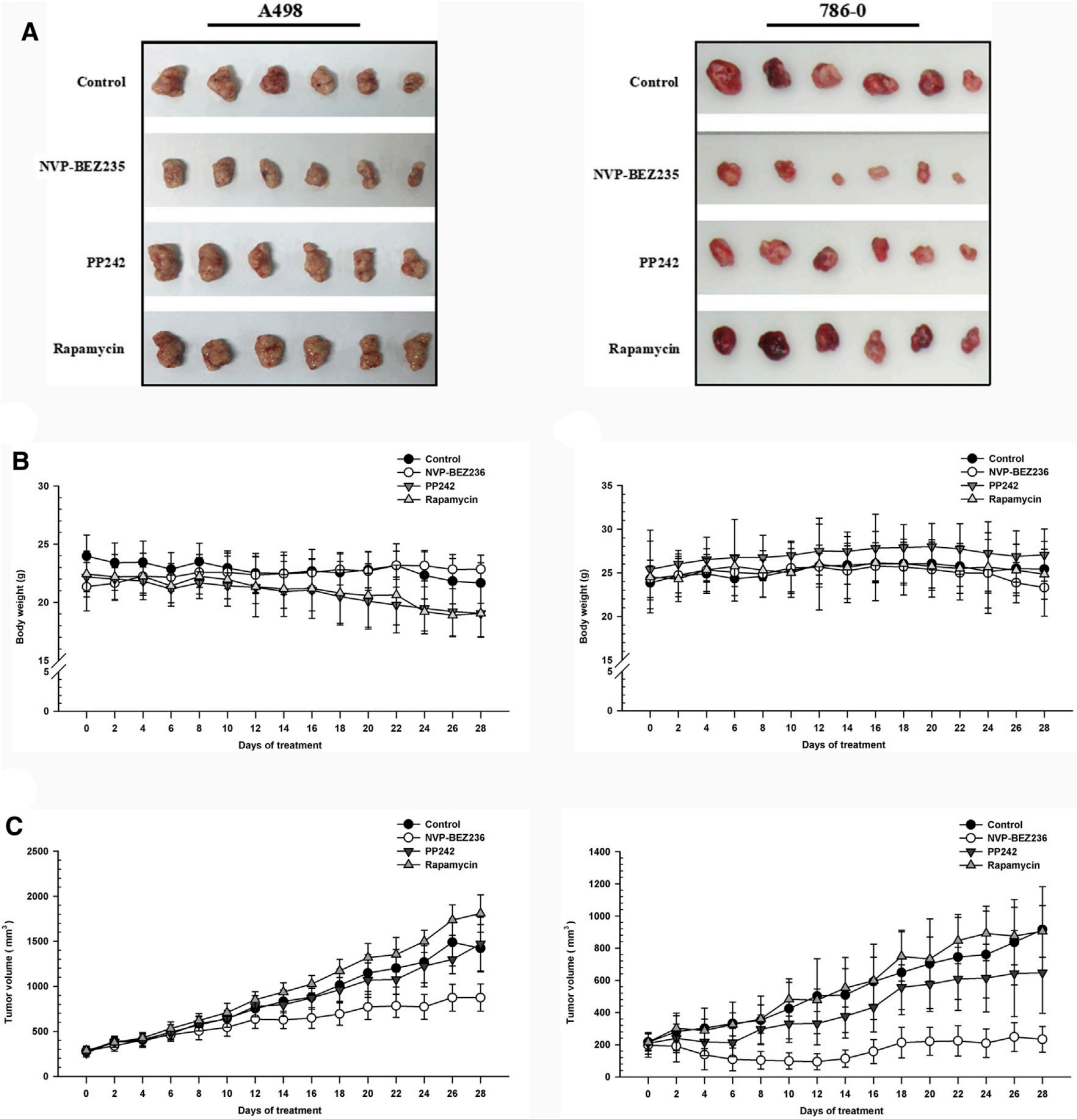
Suppression of tumor growth by NVP-BEZ235, PP242 and Rapamycin *in vivo*. Subcutaneous RCC tumors were established by injection of 786-0 or A498 cells into nude mice. The treatment with NVP-BEZ235, PP242, and Rapamycin (15 mg/kg) started after the tumor size reached around 300 mm^3^. All mice were sacrificed 28 days post injection. **(A)** Tumors were excised from the flank of these mice (*n* = 6/group). **(B)** Body weight and **(C)** tumor volume of A498 and 786-0 xenografts were measured periodically. A group of untreated mice served as control. Results are means ± SD of 6 different mice.

**FIGURE 8:**
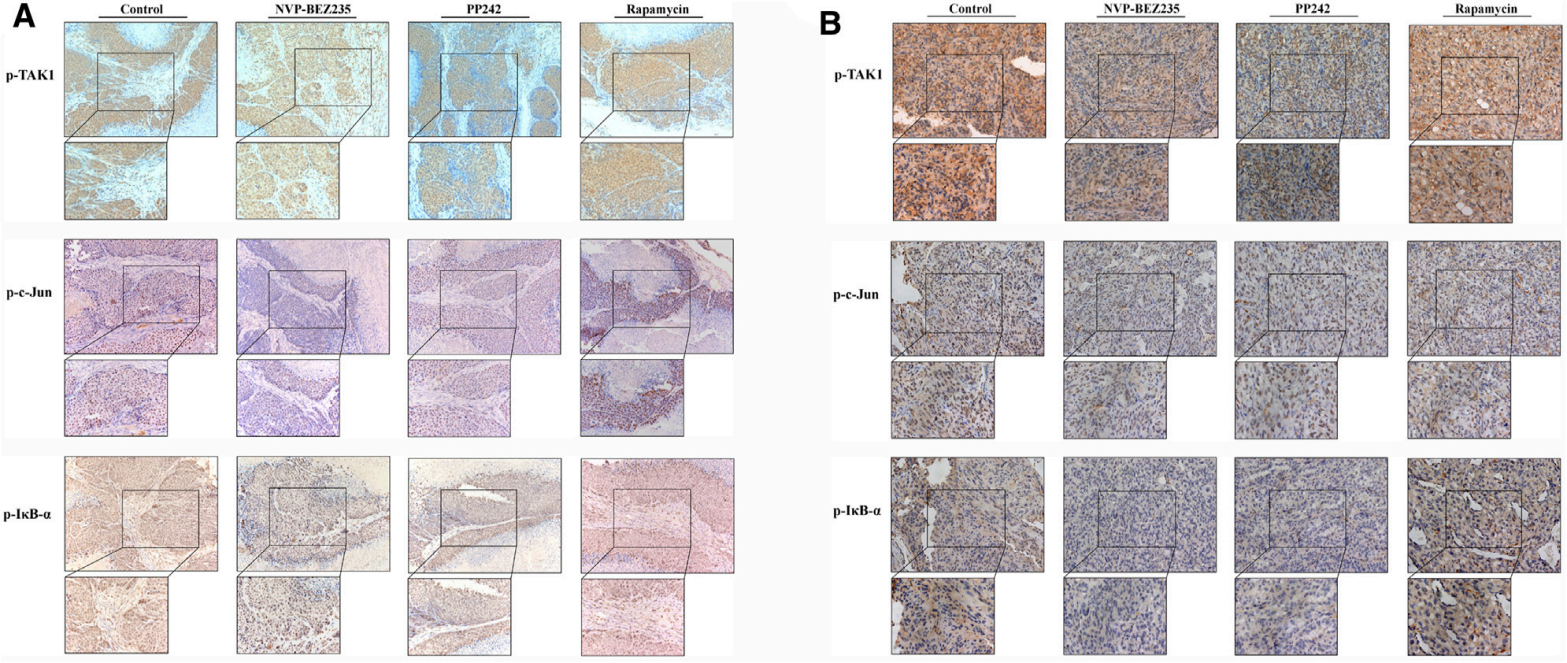
Inhibition of the activity of TAK1, c-Jun, and IκB-α in RCC xenografts in response to treatment by NVP-BEZ235, PP242, and Rapamycin. Sections of all xenograft tumor tissues formed by **(A)** 786-0 and **(B)** A498 renal cancer cells were examined by immunohistochemical staining using anti-p-TAK1, anti-p-c-Jun, and anti-p-IκB-α antibodies. Representative images were taken at a magnification 200× (inset 400×). A group of untreated mice served as control.

To determine the *in vivo* efficacy of NVP-BEZ235, PP242 and Rapamycin against human renal cancer, nude mice bearing 786-0 or A498 tumor xenograft were treated daily with these compounds as described in Methods. As shown in Figure 7B, no significant change was observed in body weight in 786-0 xenograft model within each group, while the mice administrated with PP242 and Rapamycin showed decreased body weights as compared with the weights of the controls in A498 xenograft mouse model. In general, the tumors induced by 786-0 or A498 cells injection were both most sensitive to treatment with NVP-BEZ235, followed by PP242 (Figures 7A,C). At the end of the experiment, the tumor volumes of the nude mice bearing 786-0 and A498 cells were respectively reduced by 75 and 44.6% with treatment of NVP-BEZ235, while PP242 treatment reduced tumor volumes by only 31% in 786-0 xenografts and 10.5% in A498 xenografts. Instead, Rapamycin displayed modest anti-cancer properties *in vivo* or, unexpectedly, even slightly promoted tumor growth at the late stage in mice bearing A498 tumor xenograft. By immunohistochemical staining, we demonstrated that NVP-BEZ235, and PP242 caused decrease in phosphorylation of TAK1, c-Jun and IκB-α in tumor tissues, indicating that the *in vivo* anticancer activity of NVP-BEZ235 and PP242 against RCC also depends on TAK1/JNK and TAK1/IKK signaling pathways (Figure 8). In agreement with the *in vitro* results, these decrease was more pronounced in NVP-BEZ235-treated xenograft model, while Rapamycin showed no inhibitory effects on phosphorylation of these molecules.

## Discussion

A major reason that highlights the importance of targeting mTOR in RCC relies on the observation that mTOR signaling pathway is activated in RCC, contributing to cancer development (Porta et al., 2014). Rapamycin is the first identified highly selective mTORC1 inhibitor. By binding to the FKBP12/rapamycin-binding (FRB) domain of mTORC1, Rapamycin induces the dissociation of Raptor from mTORC1, and subsequent a loss of contact between mTORC1 and its substrates (Xiong et al., 2016; Costa et al., 2018). However, the clinical outcomes of Rapamycin have been poor in RCC patients due to the reactivation of PI3K/Akt via S6K1-dependent feedback loops (Saxton and Sabatini, 2017). Then some selective ATP-competitive mTOR kinase inhibitors were developed as alternatives to the Rapamycin-based therapies. These inhibitors such as PP242 can bind to the active sites of both mTORC1 and mTORC2, and then inhibit phosphorylation of their substrate S6K1 and Akt, thereby achieving the dual target of mTOR function and Akt feedback activation (Zhang et al., 2016). Sure enough, PP242 showed better anticancer effects than Rapamycin in renal cell carcinoma (Maru et al., 2013). Meanwhile, based on the high sequence homology within the catalytic domains between PI3K and mTOR, another generation of dual PI3K/mTOR inhibitors were developed. These drugs could directly block not only mTORC1 and mTORC2, but also PI3K activity (Yang et al., 2019). This class of drugs also has been shown to yield better results than Rapamycin. As a major member of dual PI3K/mTOR inhibitors, NVP-BEZ235 has undergone Phase I/II trials for the treatment of some malignancies including renal carcinoma (Mayer and Arteaga, 2016).

In present study, we evaluated and compared the anticancer activity of Rapamycin, PP242, and NVP-BEZ235 in renal cell lines *in vivo* and *in vitro*. It was demonstrated that Rapamycin, PP242, and NVP-BEZ235 all suppressed cell proliferation and invasion, and induced apoptosis in RCC cell lines. Moreover, the inhibitory effects of these compounds basically decreased in the order of NVP-BEZ235 > PP242 > Rapamycin. Likewise, the reduction in tumor size was most prominent in NVP-BEZ235-treated xenograft mice, while Rapamycin unexpectedly failed to inhibit tumor growth. In addition, our data indicate that the three compounds induced G1 cell cycle arrest in RCC cells, consistent with previous studies (Calero et al., 2017). It can be inferred that the growth-suppressive effects of NVP-BEZ235, PP242, and Rapamycin is due to the apoptosis and cell cycle arrest.

In view of the involvement of PI3K/Akt/mTOR pathway in the inhibitory action of Rapamycin, PP242, and NVP-BEZ235, we investigated the phosphorylation of two key molecules (Akt and mTOR) in this pathway. As expected, NVP-BEZ235 simultaneously blocked Akt phosphorylation and mTOR activation in all three RCC cell lines, while PP242 directly inhibited mTOR activity and subsequently prevented activation of Akt. Nevertheless, only reduced levels of phospho-mTOR were present in Rapamycin-treated RCC cells. Rapamycin had no stable effects on the down-regulation of phospho-Akt in 786-0 and UOK121 cells, and even induced feedback activation of Akt in UMRC6 cells.

In an attempt to elucidate the regulation of PI3K/Akt/mTOR signaling in RCC, we examined the activation of SPOP (also known as E3 ubiquitin ligase adaptor), the mutation of which has been shown to be positively associated with activation of PI3K/Akt/mTOR in human cancers such as prostate and colorectal cancers (Blattner et al., 2017; Xu et al., 2015). In three RCC cell lines treated with NVP-BEZ235 and PP242, the attenuated SPOP expression occurred in parallel with inactivation of Akt and mTOR, while Rapamycin had only modest effects on SPOP expression in spite of a transient lowering. In total, these findings support the view that the distinct inhibition of PI3K/Akt/mTOR signaling by Rapamycin, PP242, and NVP-BEZ235 was partially responsible for their varying inhibitory effects in human renal cancer cells. Furthermore, SPOP-mediated PI3K/Akt/mTOR signaling pathway was more significantly altered by NVP-BEZ235 than PP242.

Despite clear evidence that activation of PI3K/Akt/mTOR pathway contributes to RCC development and progression, inhibition of mTOR and/or PI3K did not provide effective and long-lasting anticancer benefits (Alzahrani, 2019). In this context, combination therapies with other anticancer agents might be an effective strategy to improve the clinical outcome of these mTOR inhibitors in RCC patients. For instance, the combination of NVP-BEZ235 and cisplatin produced synergistic antitumor effects on drug-resistant non-small cell lung cancer cells (Zhu et al., 2020). Therefore, it is important to understand the precise mechanism of these mTOR inhibitors, so as to optimize the combination therapy.

As one MAPK family member, JNK has been identified as a pro-tumorigenic factor in many cancer types. Its activity is mainly dependent on the phosphorylation of AP-1 family members, which was confirmed here in RCC cells by repression of AP-1 activity in the presence of JNK inhibitor (Oh et al., 2017). The AP-1 family, composed of c-Fos and c-Jun proteins, is characterized as inducible transcription factors in signal transduction processes. Activated JNK (p-JNK) triggers phosphorylation of c-Jun at sites in the N-terminal domain, which induces homodimerization of c-Jun. Subsequently, c-Jun/AP-1 binds to TPA-response elements (TRE) elements in the presence of co-factors, and activates transcription of genes responsible for cell proliferation, differentiation, apoptosis and migration (Papavassiliou and Musti, 2020). Previous study has shown that JNK/AP-1 pathway is activated in RCCs (An et al., 2013). Consistently, we here observed that phosphorylation of c-Jun was significantly overexpressed in RCC tissues. Moreover, both NVP-BEZ235 and PP242 inhibited c-Jun phosphorylation and AP-1 activity in RCC cell lines, while Rapamycin only inhibited AP-1 activity but not induced stable inactivation of c-Jun in 786-0 and UOK121 cells. These findings demonstrated the involvement of JNK/AP-1 signaling in the antitumor action of NVP-BEZ235 and PP242 rather than Rapamycin. The possible mechanism is that NVP-BEZ235 and PP242 suppress JNK activity, decrease c-Jun phospholation, block AP-1-DNA binding formation and finally induce alteration of gene expression, which needs further study.

TAK1 is a serine/threonine protein kinase of the MAP3K family. Several lines of evidence suggest that TAK1 possesses the ability to activate the downstream JNK/AP-1 and IKK/NK-κB pathways, leading to RCC progression (Hui et al., 2018; Aashaq et al., 2019). Consequently, in present study, AP-1 activity was also successfully blocked with the specific TAK1 inhibitor in RCC cells. Furthermore, NVP-BEZ235 and PP242 elicited a sustained decrease in TAK1 phosphorylation in RCC cell lines, along with inactivation of IκB-α in 786-0, and UOK121 cells. Of note, we similarly proved the elevated expression of phospho-IκB-α in RCC tissues. Based on these findings, it could be speculated that besides PI3K/Akt/mTOR pathway, TAK1-dependent inactivation of JNK/AP-1, and IKK signaling may be also responsible for the inhibitory effects of NVP-BEZ235 and PP242 against renal cell carcinoma but not Rapamycin. This possibly provided another anticancer mechanism of these compounds. Support for this idea comes from the *in vivo* experiment, where treatment of 786-0 and A498 cells xenograft-bearing mice with NVP-BEZ235 and PP242 resulted in significant inhibition of TAK1, c-Jun, and IκB-α activities. As expected, these inhibitory effects by Rapamycin were modest and transient. Meanwhile, we here demonstrated the distinct regulation of PI3K/Akt/mTOR and TAK1 signaling between UMRC6 and the other RCC cell lines, which might be explained by the cell type specificity and require further research.

It is worthy of note that the JNK and NF-κB signaling pathways are also involved in ferroptotic cell death (Ye et al., 2019; Schmitt et al., 2021). Ferroptosis has been recently proved to be critical in the modulation of tumor growth and progression in some cancer types among which renal cell carcinoma is particularly susceptible to ferroptosis. It is known that the ferroptosis process involves accumulation of reactive oxygen species (ROS) from lipid peroxidation within the cell. Furthermore, there is increasing understanding that ROS-mediated signaling cascades include MAPKs and NF-κB (An et al., 2019). Thus, we speculated that the inhibition of JNK/AP-1 and IKK signaling by NVP-BEZ235 and PP242 may induce ROS-dependent ferroptosis, thereby contributing to their anti-tumour effect on RCC cells. Li et al. constructed a nanoparticle carrying Rapamycin and ferroptosis-inducer erastin, which elicited robust ferroptosis-induced cytotoxicity *in vivo* (Li et al., 2019). They proved that Rapamycin played an important role in strengthening the ferroptotic cell death. Considering the high sensitivity of urinary tract tumors to programmed cell death including ferroptosis, we believe that combination of ferroptosis-inducer with ATP-competitive mTOR kinase (such as PP242) or dual mTOR/PI3K (such as NVP-BEZ235) may be a new strategy for treating RCC. Therefore, there is need for further investigation into the correlation between ferroptosis and anti-cancer mechanism of these mTOR inhibitors.

**FIGURE 9:**
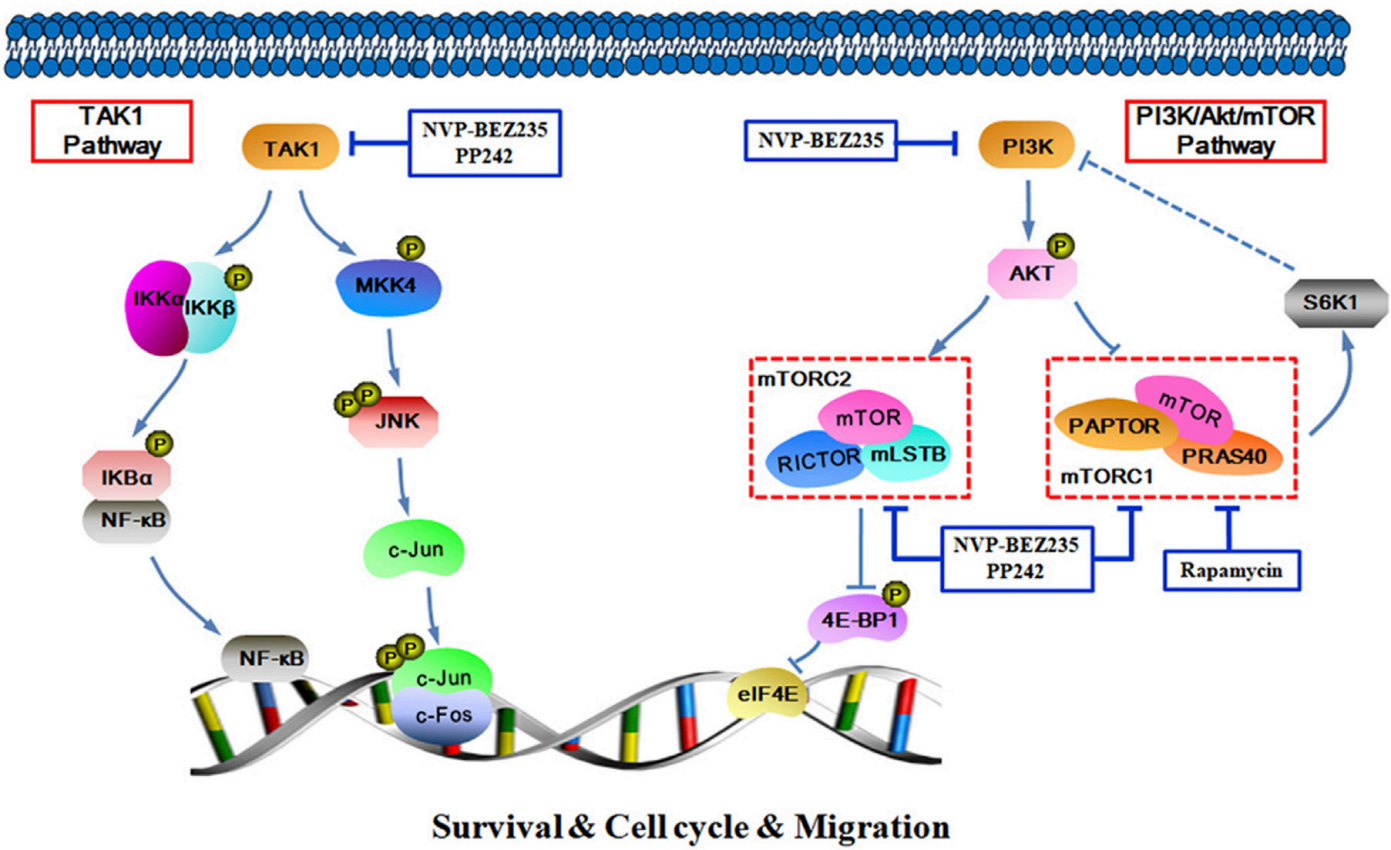
Schematic figure of biochemical pathway whereby NVP-BEZ235, PP242, and Rapamycin lead to inhibition of RCC cell proliferation, survival, and invasion.

In summary, the data presented here demonstrate that NVP-BEZ235, PP242, and Rapamycin all exhibited anti-proliferative, pro-apoptotic and anti-invasive effects against RCC cells, and the inhibitory activity decreased in an order of NVP-BEZ235 > PP242 > Rapamycin. Furthermore, in addition to PI3K/Akt/mTOR signaling pathway, TAK1-dependent JNK/AP-1 and IKK pathways may also be important in the anticancer action of NVP-BEZ235 and PP242 against RCC (Figure 9). These findings revealed a new mechanism for the superior anticancer efficacy of NVP-BEZ235 compared to PP242 and Rapamycin in RCC, providing experimental basis for its clinical application.

## Data Availability

The original contributions presented in the study are included in the article/Supplementary Material, further inquiries can be directed to the corresponding authors.

## Supplementary Material

Supplementary Figure 1 The alteration of Akt and mTOR phosphorylation detected by Western blot from the Figures 4A,B were quantified by densitometric analysis and corrected by using data of total Akt and β-actin protein content. All value are means ± SD of three replicates. **p* < 0.05, ***p* < 0.01 versus the corresponding control (DMSO or 0 nM) group.

Supplementary Figure 2 The alteration of SPOP expression and TAK1 phosphorylation detected by Western blot from the Figures 4C, 6D were quantified by densitometric analysis and corrected by using data of β-actin and total TAK1 protein content. All value are means ± SD of three replicates. **p* < 0.05, ***p* < 0.01, versus the corresponding control (DMSO or 0 nM) group.

Supplementary Figure 3 The alteration of c-Jun and IκB-α phosphorylation detected by Western blot from the Figures 6E,F were quantified by densitometric analysis and corrected by using data of total protein content. All value are means ± SD of three replicates. **p* < 0.05, ***p* < 0.01 versus the corresponding control (DMSO or 0 nM) group.
