# Supplementary figures and images for: NVP-BEZ235 Inhibits Renal Cell Carcinoma by Targeting TAK1 and PI3K/Akt/mTOR Pathways
Source: Front Pharmacol. 2022 Jan 10;12:781623. doi: 10.3389/fphar.2021.781623 (PMC8784527; doi:10.3389/fphar.2021.781623)

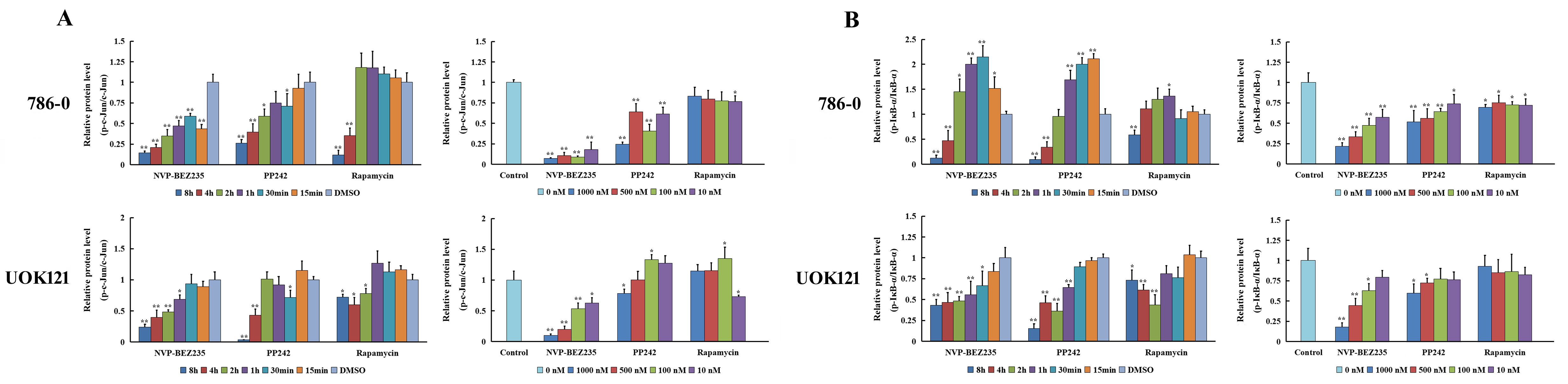

Supplement: Supplementary file 1 [file Image3.JPEG]

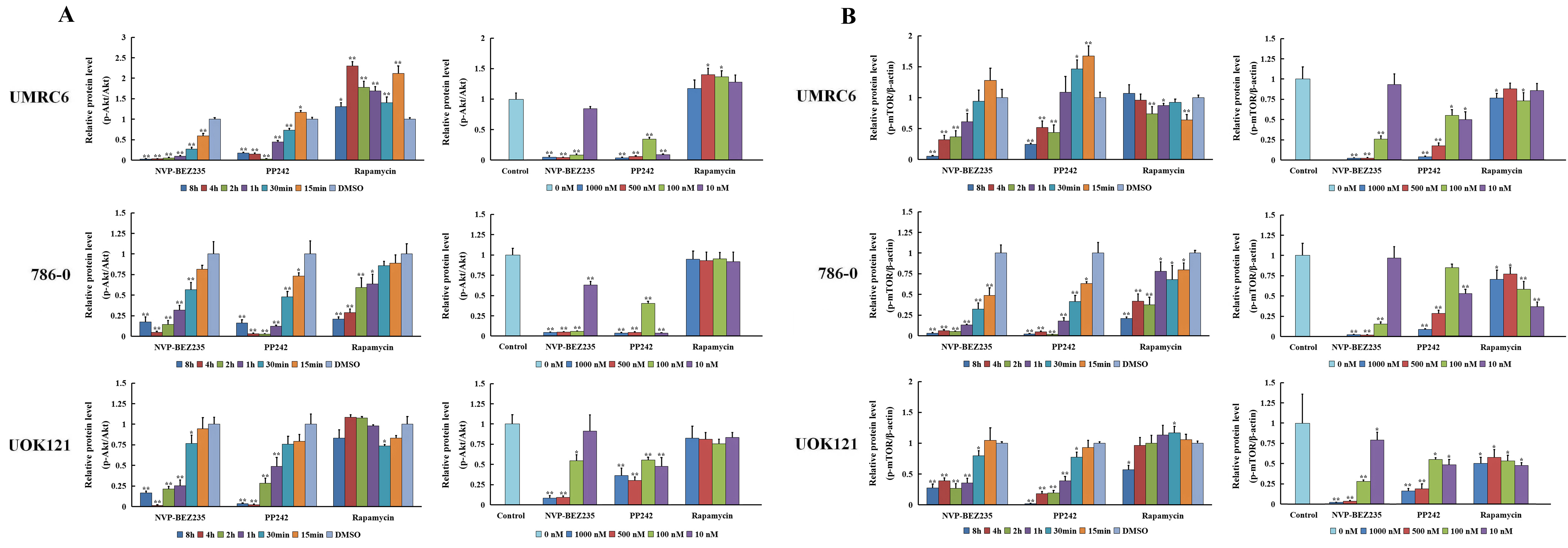

Supplement: Supplementary file 2 [file Image1.JPEG]

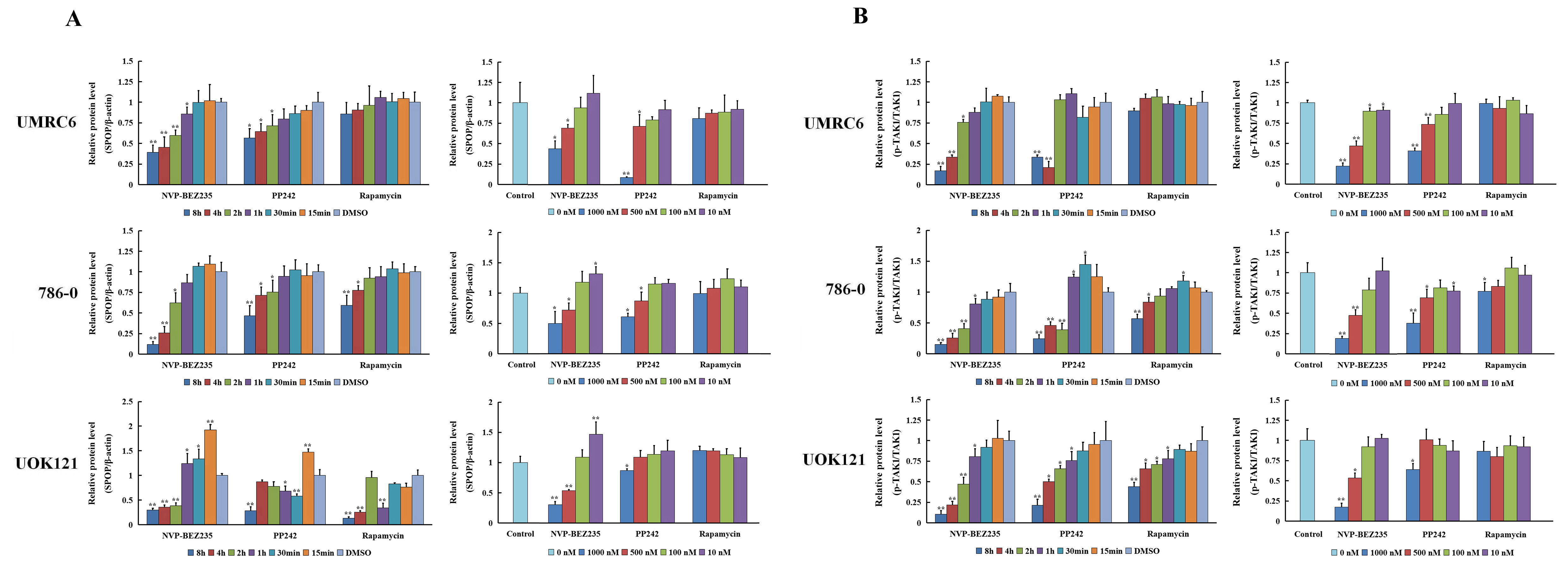

Supplement: Supplementary file 3 [file Image2.JPEG]
